# Effect Evaluation of Mental Nursing in Nursing of Young Cancer Patients Based on Big Data

**DOI:** 10.3389/fpubh.2022.888183

**Published:** 2022-04-29

**Authors:** Yuanpeng Ren

**Affiliations:** School of Nursing, Nanjing Medical University, Nanjing, China

**Keywords:** psychological approach, nursing, cancer patients, quality of life, big data, blockchain, artificial intelligence

## Abstract

The field of mental health nursing has always been special, dynamic, and flexible in terms of enhancing individuals' quality of life. Mental health nursing is all about caring for individuals with mental health disorders with the aim of helping them recover from illness, thereby improving their living conditions. Health Education of mental nursing emphasizes on providing special care to patients and optimally fulfills the clinical outcomes of the patients. It has been widely accepted that mental illness negatively influences the well-being of individuals, irrespective of their age, origin, demography, social status, and economic status. Young patients diagnosed with cancer report that they have a poor quality of life, as they undergo several physical and psychological needs, especially after the surgery and other cancer-related treatments. Thus, this study aims to examine the effect of mental health nursing on nursing young patients with cancer, based on the big data. Thus, the study conducts longitudinal analysis of the data gathered from the young patients with cancer, monitored by mental health nurse, with advanced practice nurse (APN) interventions. Results of the study stated that APN interventions positively impacted the physical as well as psychological needs of the research subjects. Mental nursing interventions resulted in positive clinical outcomes of the patients.

## Introduction

Young patients who suffer from cancer seem to experience exceptional challenges in leading a quality life. Post-surgeries and cancer treatments have a negative impact on the living conditions of patients with cancer. These patients will have to face several issues, such as difficult in establishing and maintaining relationships with fellow beings, infertility, feeling extremely dissatisfied with their body image, being dependent on others for physical needs and psychological needs, and many others. Broadly speaking, the living standards of an individual with an emphasis on health are referred to as a broad concept that covers both negative and positive aspects of life (1). Furthermore, quality of life is a multidimensional concept that encompasses health evaluations of individuals in a subjective manner. Comprehensively speaking, young patients with cancer are more likely to develop negative behaviors, such as drinking, chain-smoking, and having unprotected sex. However, these behaviors are not observed in older adult patients though (2). Seminal contributions have been made by researchers in examining the health behaviors of young patients with cancer. It was found that lack of nutrition, lack of required physical activity, lack of safety when exposed to the sun, and getting addicted to pain relievers are some of the risky health behaviors commonly developed among young patients with cancer (3).

Young patients with cancer generally fall under the age group of 15–39 years. The National Cancer Institute records the lowest age of cancer-affected patients as 12 years and the highest as 45 years (4). The majority of the patients who fall under the lower age bracket are moving toward independence and monotony, desire to lead their social and financial life independently, ready to take up social and financial responsibilities, ready to take up work, and eventually set up personal goals and values for life (1). An individual getting diagnosed with cancer at this age can lead to severe disruptions in their life. Such patients' quality of life will be badly affected as they will fail to fulfill various milestones in the phase of life at each developmental stage. While, older adult patients with cancer may be distressed as a result of interventions into family relationships, intimate or romantic relationships, and the stacking of financial burdens (5, 6).

With the advancements in the field of technology, innovative cancer treatments have helped medical professionals improve the survival rates of patients with cancer, early identification of late effects, deal with issues of survival, and enhance the quality of life (7). Recent studies have tried to focus on the factors that affect the patients' quality of life. In general, young patients with cancer would require special care and attention because they face unique experiences that older adult patients with cancer do not (8, 9). Young patients with cancer tend to have different cognitive, psychosocial, and emotional developmental stages when compared witholder adult patients. Henceforth, these differences in their developmental stage will certainly influence their illness and also their experiences. Past studies confirmed that young patients with cancer are recognized as a high-risk group. It was also found that young patients with cancer had a good quality of life and experienced more survival improvements than older adult patients (10, 11).

Young patients with cancer, in general, receive aggressive treatments that are complex in nature. Some of the treatments include surgery, hysterectomy, bilateral salpingo-oophrectomy, resection of surrounding tissue, lymph node dissection, and omentectomy. Sometimes, patients will have to initiate chemotherapy (12). An individual getting diagnosed with cancer at this early age can lead to severe disruptions in their life. Such patients' quality of life will be badly affected as they fail to fulfill various milestones in the phase of life at each developmental stage (13). While, older adult patients with cancer might experience distress as a consequence of interventions to family relationships, intimate or romantic relationships, the stacking of financial burdens. In addition, it was found that nursing professionals were successful in developing hope among patients with cancer, as they carried right potential among themselves to care for patients with cancer (14). Healthcare professionals can literally transfer the element of hope to those who are suffering from chronic diseases, such as cancer, and hence, they can work together on activities focusing on hope. Past studies indicated that patients with cancer exhibited high levels of hope with a better quality of life after the interventions of healthcare professionals (15–17). One of the studies discussed that hope therapy, mindfulness, and other behavioral components helped patients with cancer to instill a positive mind. Without any doubt, such interventions aided the patients to have a better quality of life, especially after 6–7 months of interventions. Despite all this, the effectiveness of healthcare professional's inventions has remained a controversial topic until date, which became the motivation to carry out this research. To improve the quality of treatment, quality of results, and quality of patient's life, this study aims to shed light on the physical and emotional needs of patients (18–20). In addition, the study stresses the importance of providing special care to the young patients with cancer, which will help them understand the challenges faced by the young group. This article follows the layout given below. Section Literature Review presents the examination of available literature review. Section Methodology discusses the methodology adopted in the study and the ways to gather data. Section Findings and Discussion presents the prominent findings derived from the study followed by discussion. Section Conclusion presents the conclusion of the article.

## Literature Review

The literature review shows that patients with cancer generally face issues physically, mentally, and socially, which will result in experiencing a poor quality of life. It was found that cancer treatments can badly affect an individual's nutrition, leading to several other chronic diseases, such as heart attacks. Additionally, patients with cancer tend to have mood disturbances that develop as part of chemotherapy treatments. In fact, these factors certainly affect the hope of an individual. Certain studies identified that the element of hope was higher among those patients who experienced lower levels of anxiety and depression, along with higher levels of social, emotional, and financial support (20–22). Therefore, such patients could have a better quality of life when compared with those who have higher levels of anxiety and depression and low levels of social, emotional, and financial support.

Apart from the above, the physical appearance changes of a cancer patient look scary and ill-looking, as they include weight loss, hair loss, weight gain, and other stretch marks associated with the previously mentioned reasons. Young patients with cancer seemed very disturbed and concerned about their hair loss, weight gain, and weight loss. Most importantly, young patients with cancer considered themselves to look ugly and different after the treatments than prior to them. These reasons affected their minds and lives, which in turn negatively impacted their quality of life. Sometimes, the patients even felt traumatic due to hair loss and eventually started reporting stress during interactions with other individuals. They were constantly threatened by their own body image (23–26). Few other studies identified memory loss, disabilities with learning, disorders related to attention, and others as part of the late physical effects. The above factors will certainly impact the performance at work and at schools.

Some of the patients even face sexual problems, fertility problems, reproductive problems, obesity problems, and so on as a part of cancer treatment. The capability of reproduction is also reduced in younger patients. However, cancer survivors do have the ability to reproduce in rare cases. Cancer is one of the deadly diseases and it requires high levels of care, love, and attention. Due to the fragmentation and lack of coordination of healthcare professionals, patients with cancer feel extremely low and thus it impacts their effectiveness of nursing (21, 27, 28). Prior research suggests that physical exercises enhance the strength of those patients who are specifically suffering from lung cancer. Additionally, it was found that intervention by nurse led clinics, by means of telephonic conversation, significantly improved the level of hope among the patients during the various cancer treatments. It becomes the responsibility of a nursing professional to provide extensive help, care, attention, and love to those patients who have been diagnosed with cancer. It is their essential goal to provide special care, love, and attention to those who are struggling from having a quality life. There is a considerable amount of literature on implementing different types of nursing interventions to instill a hope factor among patients with cancer (29). These studies also examined the effectiveness of nursing interventions to foster hope among patients with cancer.

Most of the studies observed that interventions of nursing professionals promoted the mental well-being, psychological well-being, and emotional well-being of patients with cancer, thereby reducing their levels of anxiety and depression. Since hope is the only element that instills confidence among people to lead a better quality of life, it is considered as the crucial life-changing element. Previous research showed that nursing professionals were successful in developing hope among patients with cancer as they carried right potential within themselves to care for patients with cancer. Healthcare professionals can literally transfer the element of hope to those who are suffering from chronic diseases, such as cancer, and hence, they can work together on activities focusing on hope (1, 11). Previous studies indicated that patients with cancer exhibited high levels of hope with a better quality of life, after the interventions of healthcare professionals. One of the studies discussed that hope therapy, mindfulness, and other behavioral components helped patients with cancer instill a positive mind. Without any doubt, such interventions helped the patients to have a better quality of life, especially after 6–7 months of interventions. Despite all these, the effectiveness of the healthcare professional's inventions remained a controversial topic until date (6, 27). Several studies identified sleeping disorders as one of the major symptoms frequently reported by patients with cancer (22, 30).

People who underwent chemotherapy faced multiple problems with regard to the quality of sleep. Young patients with cancer faced similar kind of problem. Youngsters found it very difficult to fall asleep, remain asleep, and even regain sleep, especially when they were diagnosed with cancer. Due to this, the alertness decreased among them, and they started sleeping during the daytime. Other symptoms reported in association with sleeping disorders were pain, vomiting, nausea, thirsty, hungry, anxiety, and depression. During the time of diagnosis, the majority of the patients faced extreme pain, and they remarked that it was extremely severe (31–33). However, a few patients experienced moderate levels of pain. Ultimately, the pain-affected the individual's living standards, which ultimately lead to death and infection. When the patients are hospitalized for treatment, the pain increases, which leads to anxiety, depression, and nervousness. However, there are very limited interventions by healthcare professionals on pain (8–10, 34). Existing literature on patients with cancer also discussed the difficulties or challenges they face on a daily basis. Physical symptoms caused by cancer treatments were discovered to affect all people of all ages equally with varying degrees.

Young patients find these physical symptoms extremely odd, and hence they affect their living standards of patients. Commonly identified symptoms are eating disorders, pain, sleeping disorders, fatigue, and changes in physical appearance (14, 17, 28). Nevertheless, the most frequently reported physical symptom is fatigue, which is experienced during the diagnosis period, hospitalization period, and recovery period. Additionally, patients reported that fatigue disrupted their routines, at least for the subsequent 2 years after receiving the treatment (11, 14, 18). These symptoms caused extreme stress on the lifestyle of patients with cancer, especially at social, psychological, and emotional levels. In addition, different lifestyle activities, such as socializing with people, mild to moderate physical activities, and studying can cause fatigue. However, sometimes a lack of physical activity or boredom can also lead to fatigue. Only few studies aimed to assess the impact of interventions to manage fatigue. Similarly, several studies tried to examine the relationship between big data and nursing science and found that it is multidimensional. Since big data is more accessible and unique, the usage of this technology to enrich nursing is widely considered as a vibrant thought process (33).

Big data technology can replace traditional data sources, such as patient reports, survey measures, and symptoms status. By doing so, the nursing professional can use intervention strategies to understand the experience of the patients. Some of the big data technologies include the usage of biosensors, usage of mobile applications, different analytical platforms, and so on. Hence, big data is considered as an analytical resource, leading to innovative pathways toward achieving new knowledge (33). Additionally, big data technology allowed the professionals to analyze and interpret the data saved in the database at their convenience, so it is easier to connect. The nursing field is all about understanding reports, storing the reports, structuring the reports, and then coming up with a solution to deal with the gathered data. Thus, data become crucial for nursing professionals, especially when they deal with patients with cancer.

## Methodology

Young women who were suspected or diagnosed with cancer were identified by one of the cancer centers in the North. The study has inclusion and exclusion criteria based on which the participants were selected. The inclusion criteria covered the following: individuals identified with cancer during the primary stage; individuals planning to begin their chemotherapy treatment; individuals who have been discharged after their first surgery; individuals who are above 22 years of age; individuals living in the northern part of India; and individuals who have been diagnosed with cancer for at least 6–7 months. Individuals who were not diagnosed with cancer were excluded from the study. People who have passed away were also excluded. A nurse recruiter identified the research subjects suitable to the study during the period of January 2017 to July 2020. Baseline questionnaires were administered to the Project Directors of the selected hospitals and obtained consent from them to collect the data and perform further analysis. As part of data collection process, the nurse professionals conducted in-person interviews with the selected patients at their residences. With the help of trained research assistants, the nursing recruiters conducted the interviews to obtain the baseline data. Subsequently, the patients who gave their consent were randomly assigned into control groups or interventions with the help of sealed envelope technique. Overall, 280 women were considered as the final participants, who did have the eligibility to be part of the research. Of 280, 60 dropped out of the reason for discharged as they were not scheduled for the next cancer treatment. Out of 220 respondents left out, 150 of them enrolled themselves, constituting 70% of the response rate. Of 150 enrolled participants, the baseline data shared by 5 of them were incorrect and thus they were excluded. Only 123 participants focused on completing the outcome measures at the estimated time of 6 months. Furthermore, 22 women failed to complete the study as 19 of them died during the survey, 2 of them were in a state of bad condition, and one of them was extremely anxious and overwhelmed.

The researcher adopted advanced practice nurse (APN) interventions, who took special care of those patients who were grouped into control nursing interventions. The duration of the APN interventions was 6–7 months, during which the nursing professionals trained the patients to develop and maintain self-management skills. This intervention also encouraged the selected participants to actively participate in taking decisions that positively impacts their following treatments, such as chemotherapy. Managing and monitoring symptoms, balancing emotions, educating patients, coordinating with resources and referrals, providing nursing care were some of the APM intervention activities carried out in the research. On understanding the needs and priorities of a patient, the nursing professionals came up with a plan to implement intervention strategies that was jointly developed by the patient and nursing professional. The needs of patients differed from one person to another. These include medication management, nutrition, fulfilling spiritual needs, concerns about family members, issues related to sex, managing pain, and ways to deal with chemotherapy. Table 1 records the details of scheduled interventions with the control groups.

**Table 1:** Schedule of contacts for intervention and attention control groups.

**Group**	**Qualification of interventions**	**Participants**	**No of contact**	**Nature of contacts**	**Care provided to the participants**
Special nursing intervention programs	APN in oncology	64	20 total; 3 per week, month 2; 3 per month, months 3–7	9 home visits, 6 telephone calls, 4 clinic visits	Teach self-management skills, Stabilize post-surgery, Symptom management for chemotherapy side effects, Counseling and support
Psychiatric consultation–liaison nurse with advanced practice nurse interventions	APN in mental health nursing	33 of 64	1 to 3 additional contacts	2 clinic visit/home visit for evaluation, 1 telephone follow-up	Psychiatric evaluation for high emotional distress, Identification of resources
Attention control	Research assistant	61	9 total; 2 per week, month, 2 per month, months 3–6	1 home visit; 1 Contacts 3–9: telephone calls	Symptom management for chemotherapy

Using a distress thermometer, the emotional distress of the patients was screened at the baseline assessment. Those patients, who scored > 4 or 4 on the distress thermometer, were recommended to receive Psychiatric Consultation from Liaison Nurse (PCLM). This evaluation examined the emotional needs of the patients to identify the psychiatric disorders, if any, as proposed by National Comprehensive Cancer Network. The nursing professionals and liaison nurse jointly reviewed the planned care along with the patient. Even the symptom management kit was distributed to the patients, which covered 15 and above symptoms that are commonly experienced by patients with cancer. This kit also covered the details of the symptoms, different strategies to deal with the symptoms, and the right time to contact the doctor. Trained research assistants took care of the patients who were put in the control groups. These assistants had adequate knowledge of the symptom management kit. All this assistance took place at the participant's house, where they explained the advantages and uses of the symptom management kit. These trainers also implemented different strategies to manage the symptoms of cancer. Besides, patients who had queries out of the baseline assessment were encouraged to call their oncologist. The trainers conducted a weekly visit to the patient's house, conducted weekly telephonic conversations, and made monthly telephonic calls even after they were discharged. All the telephonic conversations almost lasted for an average of 20 min.

The validity and reliability of the baseline assessment were measured by adopting standardized measures that have strong psychometric properties. These measures will help the researcher to understand the variables that are crucial to quality of life. These variables included uncertainty, symptoms of distress, symptoms of depression, and a better quality of life. A Depression Scale was implemented to measure the symptoms of depression that consists of 20 items. The range of the scale measured from 0 to 60. Ambiguity illness scale was used to measure the uncertainty factor, which ranged from 12 to 64. As the score went high, it was inferred that the level of uncertainty was higher. Symptom Distress Scale was used to measure the symptoms of distress, which has 13 common symptoms, widely experienced by patients with cancer. Consistency of the values obtained from the various scales and the result of test-retest observed that the scale was reliable and valid. Both mental and physical health of the patients were assessed using a short health survey that included 11 items. This survey helped the researcher to analyze the quality of life-based on the scores that ranged from 0 to 100.

SAS software, version 9.1, was used to analyze the data collected in the study. To analyze the demographic details of the participants, the researcher adopted descriptive statistics. Other statistical methods used in the study to measure the quality of patient's life, mean, median, and standard deviation (SD) were used during the initial baseline measure, after 1st month, after 3 months, and lastly between 4 and 6 months. Those patients who dropped out in between were not included in the data analysis process. The primary aim of the analysis was to understand the impact of nursing interventions on young patients with cancer, which also helped the researcher to understand their quality of life. Mixed effect regression models were used to examine the interventions at longitudinal level.

## Findings and Discussion

**Figure 1:**
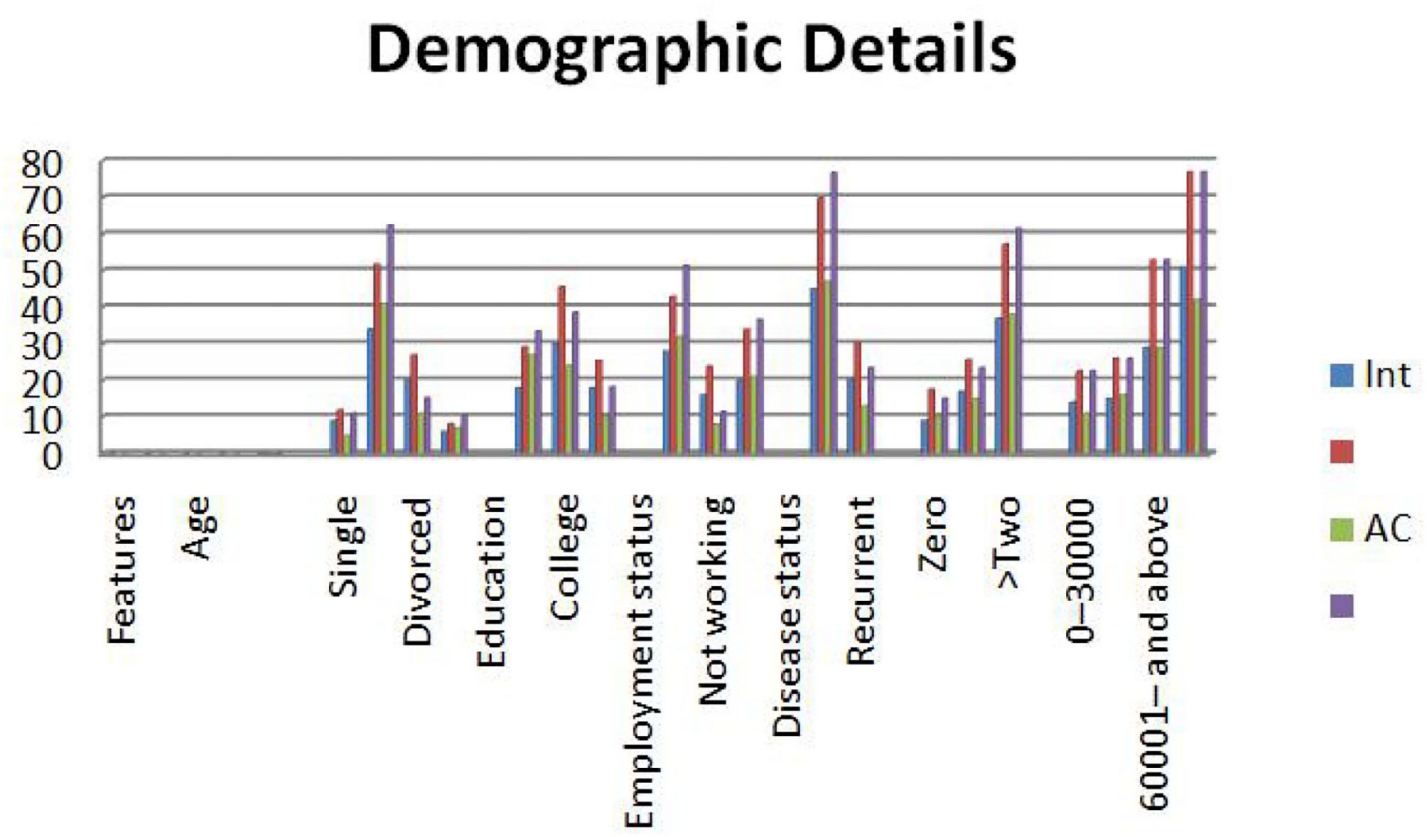
Graphical representation of demographic details.

The detailed analysis of the sample collected is recorded in Table 2 and Figure 1. Most of the participants were women who were diagnosed or suspected of having cancer. Out of 123 participants, 62% of them were diagnosed with ovarian cancer and would require chemotherapy. About 73% of the participants were newly diagnosed with cancer, and 28% had the disease on a regular basis. The results of the study state that 33% of the participants were diagnosed with cancer either in the primary stage or secondary stage. About 67% of them were diagnosed with cancer during the last stages. About 60% of the participants had more than two comorbidities. About 78% of the patients came with a family history of cancer. Most of the participants had different characteristics, even in the terms of demography and clinical symptoms.

**Table 2:** Demographic details of the patients.

**Features**	**Int**		**AC**	
	**(*****N*** **=** **62) MM (±SD)**		**(*****N*** **=** **61) M (±SD)**	
Age	57.2 (11.1)		61.23(11.7)	
DT	5.71 (2.7)		5.14 (2.8)	
	N	%	N	%
**Marital status**				
Single	9	11.9	5	11.0
Married	34	51.7	41	62.4
Divorced	20	26.9	11	15.2
Widowed	6	8.1	7	10.5
**Education**				
High school	18	29.1	27	33.5
College	30	45.6	24	38.6
Graduation	18	25.4	10	18.2
**Employment status**				
Employed	28	42.9	32	51.4
Not working	16	23.9	8	11.4
Studying	20	33.9	21	36.7
**Disease status**				
New	45	69.9	47	76.8
Recurrent	20	30.23	13	23.5
**Comorbidities**				
Zero	9	17.6	10	15.1
One-Two	17	25.6	15	23.4
>Two	37	57.3	38	61.5
**Income**				
0–30000	14	22.6	11	22.6
30001–60000	15	26	16	26
60001– and above	29	53	29	53
Family history of cancer	51	77	42	77

Table 3 presents the scores against each measures or variables, during the baseline assessment, after 1st month, after 3 months, and between 4 and 6 months. Baseline scores of the data had significant differences. Additionally, it was reported that the quality of life of patients was poor. The psychological and physical impacts of high-risk illness were adjusted in the reports. Tables 4–6 presented the results of mixed-effect regression. The coefficient measures, standard errors (SEs), and *p* of all the four waves (wave 1—baseline assessment; wave 2—after 1st month of intervention; wave 3—after 3 months of intervention; and wave 4—between 4 and 6 months of intervention) are recorded.

**Table 3:** Measure of unadjusted quality of life.

	**Baseline Assessment**		**After 1 month**		**After 3 months**		**After 6 months**	
	**Int**	**Ctl**	**Int**	**Ctl**	**Int**	**Ctl**	**Int**	**Ctl**
**Measure**	**M ±SD**	**M ±SD**	**M ±SD**	**M ±SD**	**M ±SD**	**M ±SD**	**M ±SD**	**M ±SD**
CESD	18.2, 7.5	15.1, 8.1	13.6, 7.5	11.0, 7.3	13.9, 8.3	11.1, 7.8	12.2, 8.1	8.7, 6.0
MUIS	39.4, 8.9	34.9, 9.3	35.4, 9.4	32.8, 9.5	35.1, 9.4	31.9, 9.6	31.4, 10.7	28.0, 10.7
SD	29.3, 6.8	26.9, 6.8	25.5, 7.0	23.4, 6.6	24.8, 6.4	22.2, 6.5	23.0, 6.9	20.1, 5.4
SF-12 Mental a	43.4, 10.0	47.9, 9.9	48.0, 10.8	51.2, 8.8	47.0, 9.9	51.3, 9.4	49.0, 9.9	52.9, 8.8
SF-12 Physical a	32.9, 9.2	33.5, 9.0	34.8, 8.9	37.0, 11.0	39.4, 10.0	41.3, 10.6	41.4, 11.7	44.9, 12.0

**Table 4:** Results of mixed-effect regression models with identified variables.

**Variables**	**Wave (without interactions)**	**Group (correction at baseline)**	Wave*group (interaction)
	**P-value ±se**	**P-value ±se**	**P-value ±se**
CESD	−0.08147 (<0.0001) ± 0.01345	0.1632 (0.0198) ± 0.06718	0.06421 (0.0030) ± 0.02201
MUIS	−0.03132 (0.0001 ± 0.00647	0.1234 (0.0243) ± 0.04176	−0.049042 (0.0006) ± 0.01287
SDS	−0.06267 (0.0001 ± 0.00497	0.05568 (0.1602) ± 0.04571	0.05187 (0.0022) ± 0.01298
SF-12 Mental	−0.00217 (0.6291) ± 0.00511	−0.05761 (0.0723) ± 0.02748	0.018675 (0.1245) ± 0.01354
SF-12 Physical	0.0739 (0.0001 ± 0.01420	0.09546 (0.1321) ± 0.06773	−0.07532 (0.0020) ± 0.02273

**Table 5:** Wave-variable interactions.

**Variables**	**Wave (without interactions)**	**Dose (correction at baseline)**	Wave*group (interaction)
	***P*** **-value ±se**	***P*** **-value ±se**	***P*** **-value ±se**
CESD	−0.08346 (0.0001) ± 0.01178	0.08456 (0.0292) ± 0.039045	0.03572 (0.0032) ± 0.01341
MUIS	−0.03733 (0.0001) ± 0.00782	0.04376 (0.1034) ± 0.02869	−0.03879 (0.0001) ± 0.00873
SDS	Poor model fit		
SF-12 Mental	−0.00231 (0.6521) ± 0.005034	0.05869 (0.0043) ± 0.01891	0.02378 (0.0032) ± 0.00765
SF-12 Physical	Poor model fit		

**Table 6:** Final mixed-effect regression models with identified variables.

**Variables**	**Wave (without interactions)**	**PCLN (correction at baseline)**	Wave*PCLN (interaction)
	***P*** **-value ±se**	***P*** **-value ±se**	***P*** **-value ±se**
CESD	−0.08487 (0.0001) ± 0.01257	−0.021983 (0.8450) ± 0.1495	0.01768 (0.6390) ± 0.03265
MUIS	−0.03478 (<0.0001) ± 0.00688	−0.03893 (0.5740) ± 0.05876	−0.04782 (0.0245) ± 0.02236
SDS	−0.06685 (<0.0001) ± 0.00496	0.2734 (<0.0001) ± 0.05765	−0.1345 (<0.0001) ± 0.012543
SF-12 Mental	−0.00248 (0.6373) ± 0.006721	−0.1132 (0.0176) ± 0.04765	0.06883 (0.0001) ± 0.01396
SF-12 Physical	0.07962 (0.0001) ± 0.01498	−0.1076 (0.3209) ± 0.1057	0.1935 (<0.0001) ± 0.033899

The results of mixed-effect regression models are recorded in Table 4. These results were analyzed with the interventions of nursing professionals. Table 4 results ignored the psychometric counseling liaison nurse and control groups. The levels of dose were modeled in Table 5, with moderate interventions. Table 6 recorded the results of nursing interventions on the quality of life. These nursing interventions will help the researcher to understand the difference in adjustment at baseline. Intervention terms, such as ‘group,' ‘dose,' and ‘PCLN,' were respectively used in Tables 4–6. The difference between intervention groups and its effect on the quality of life will also be understood from the tables.

**Figure 2:**
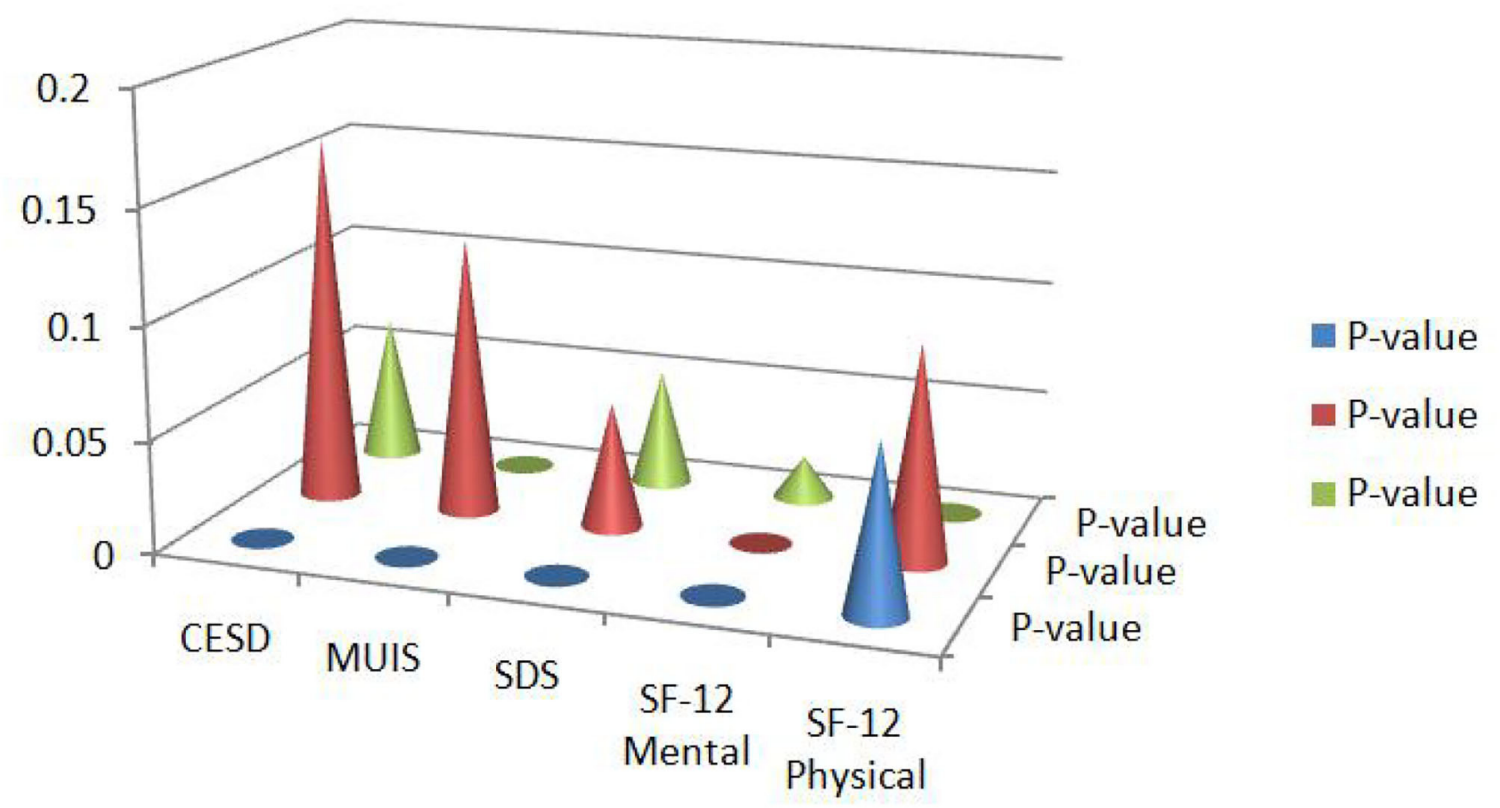
Graphical representation of *p* of the variables identified.

From the Tables 4–6, it can be inferred that the patient's quality of life improved post-conducting multiple interventions than before the interventions. The values of *p* of the baseline assessment are recorded in the first column of Tables 4–6. Similarly, slope estimates of the baseline assessment are recorded in the first column of the Tables 4–6. The mean differences in the quality of life were recorded in the second column of Tables 4–6, which are almost in line with existing studies (14, 17, 18). The main effect of intervention groups is tested using a mixed-effect regression model and the results are obtained. Tables 4–6 also recorded the key features of interactions that were in line with time. These results are recorded in the third column of Tables 4–6. From Table 4, it can be inferred that the rate of improvement in terms of having a quality life was greater for those who were in the intervention group, with a *p*-value of 0.0007, which is almost in line with existing studies (21, 22, 29, 32). Nevertheless, the attention control group also performed better in terms of having a quality life as the time progressed, with many interventions. Therefore, it can be concluded that increasing the dose highly contributed to the improvement in the quality of life, the patients were experiencing before. From the analysis, it was also observed that time was one of the factors that positively impacted the patient's quality of life, which is almost in line with existing studies (5, 7, 9, 29). Figure 2 represents the graphical representation of the values of *p* of identified variables.

## Conclusion

The present article has tested the quality of treatment, quality of results, and quality of the patient's life, and sheds light on the physical and emotional needs of the patients. The results of the study indicated that there is a relationship quality of life and nursing interventions. There is a positive relationship between dealing with interventions and big data technology, there is a positive relationship between big data and nursing, big data increases the growth and development of health sector, even during the pandemic, and technology adaptation and big data technologies will increase the easiness to deal with cancer patients even during the coronavirus disease 2019 (COVID-19) pandemic. Additionally, it was found that with nursing interventions, people were able to lead a better life than without any intervention. Thus, with nursing interventions, patients were able to cope up with the distress and thus manage their standards of living. The result of the study is only limited to this study and hence it cannot be generalized. Other limitations include: this study focuses only on patients who are either suspected to have cancer or who are diagnosed with cancer and are in different stages of cancer; study selects only female respondents for convenience; and study focuses only on young or adult patients. However, future researchers can focus on these limitations and carry research in this area. Therefore, this research is open for future work.

## Data Availability Statement

The original contributions presented in the study are included in the article/supplementary material, further inquiries can be directed to the corresponding author.

## Ethics Statement

Ethics approval and written informed consent were not required for this study in accordance with national guidelines and local legislation.

## Author Contributions

The author confirms being the sole contributor of this work and has approved it for publication.

## Funding

This work was supported by General Project of University Philosophy and Social Science Research Foundation in Jiangsu Province Research on Legal System of Palliative Care under the background of Health China Strategy (No: 2020SJA0300).

## Conflict of Interest

The author declares that the research was conducted in the absence of any commercial or financial relationships that could be construed as a potential conflict of interest.

## Publisher's Note

All claims expressed in this article are solely those of the authors and do not necessarily represent those of their affiliated organizations, or those of the publisher, the editors and the reviewers. Any product that may be evaluated in this article, or claim that may be made by its manufacturer, is not guaranteed or endorsed by the publisher.
